# Non-Human Primate Blood–Brain Barrier and In Vitro Brain Endothelium: From Transcriptome to the Establishment of a New Model

**DOI:** 10.3390/pharmaceutics12100967

**Published:** 2020-10-14

**Authors:** Catarina Chaves, Tuan-Minh Do, Céline Cegarra, Valérie Roudières, Sandrine Tolou, Gilbert Thill, Corinne Rocher, Michel Didier, Dominique Lesuisse

**Affiliations:** 1 Rare and Neurologic Diseases Research Therapeutic Area, Sanofi R&D, 91385 Chilly-Mazarin, France; catarina.chaves@sanofi.com (C.C.); Tuan-Minh.Do@sanofi.com (T.-M.D.); Celine.Cegarra@sanofi.com (C.C.); Valerie.Roudieres@sanofi.com (V.R.); 2 Translational Sciences Unit, Sanofi R&D, 91385 Chilly-Mazarin, France; Sandrine.Tolou@sanofi.com (S.T.); Gilbert.Thill@sanofi.com (G.T.); Corinne.Rocher@sanofi.com (C.R.); Michel.Didier@sanofi.com (M.D.)

**Keywords:** blood–brain barrier, brain endothelial cells, non-human primate, receptor-mediated transcytosis, transcriptomics

## Abstract

The non-human primate (NHP)-brain endothelium constitutes an essential alternative to human in the prediction of molecule trafficking across the blood–brain barrier (BBB). This study presents a comparison between the NHP transcriptome of freshly isolated brain microcapillaries and in vitro-selected brain endothelial cells (BECs), focusing on important BBB features, namely tight junctions, receptors mediating transcytosis (RMT), ABC and SLC transporters, given its relevance as an alternative model for the molecule trafficking prediction across the BBB and identification of new brain-specific transport mechanisms. In vitro BECs conserved most of the BBB key elements for barrier integrity and control of molecular trafficking. The function of RMT via the transferrin receptor (TFRC) was characterized in this NHP-BBB model, where both human transferrin and anti-hTFRC antibody showed increased apical-to-basolateral passage in comparison to control molecules. In parallel, eventual BBB-related regional differences were Investig.igated in seven-day in vitro-selected BECs from five brain structures: brainstem, cerebellum, cortex, hippocampus, and striatum. Our analysis retrieved few differences in the brain endothelium across brain regions, suggesting a rather homogeneous BBB function across the brain parenchyma. The presently established NHP-derived BBB model closely mimics the physiological BBB, thus representing a ready-to-use tool for assessment of the penetration of biotherapeutics into the human CNS.

## 1. Introduction

The blood–brain barrier (BBB) constitutes the main interface of molecular exchange between the periphery and the central nervous system (CNS) [1]. Given its extremely restrictive properties, the BBB plays a key role over the bi-directional passage of endogenous and exogenous compounds, and ultimately constitutes a formidable challenge for the entry of therapeutics into the brain [2]. In order to find new brain-specific mechanisms of transport and efficient CNS-active therapeutic alternatives, it is imperative to develop strategies to deepen the understanding of the BBB. Most in vitro BBB models derive from isolated brain capillary endothelial cells, from primary or immortalized cell line source, and rely on brain tissue from rat, mouse, porcine, bovine, and humans [3]. However, the underlying interspecies differences query the reliability and prediction capacity of most in vitro BBB models for the passage of therapeutic molecules across the human BBB and into the CNS [4,5]. More recently, human BBB models from induced pluripotent stem cell (iPSC)-derived endothelial cells have been developed, but it is still uncertain to what extent they may recapitulate the BBB and predict in vivo exposures [6].

As access to fresh human brain tissue is extremely scarce, a non-human primate (NHP)-derived BBB model may represent a valuable replacing tool for the prediction of the passage of biotherapeutics across the human BBB. To this date, apart from a commercially available model [7] very few efforts have been made to develop an NHP-derived BBB model. Furthermore, as opposed to many omics studies reported on human brain [8,9,10], there is a considerable gap in sequencing and proteomic data published regarding the primate brain endothelium. Finally, even though distribution of some drugs within the brain have been documented [11], fundamental knowledge of the BBB in distinct regional brain structures is lacking.

The objective of the present study is: (1) to establish, characterize and validate an NHP BBB model using freshly isolated endothelial cells from microvascular brain vessels of Macaca fascicularis and (2) to molecularly assess potential regional differences of the BBB across five brain structures: brainstem, cerebellum, cortex, hippocampus, and striatum. The NHP-derived BBB model was characterized molecularly, using RNA sequencing (RNAseq) tools, and functionally by focusing on its capacity to predict receptor-mediated transcytosis (RMT) phenomena via the transferrin receptor (TFRC). Through an RNAseq approach, we compared the transcriptomic profiles of freshly isolated NHP-derived brain endothelial cells (BECs) with those set in culture in order to Investig.igate their potential evolution over time and passage in vitro, along with a pairwise comparison of the transcriptomic profiles of the brain microvascular endothelial cells obtained from the aforementioned five brain regions.

In brief, in vitro selected NHP-derived BECs maintained the expression of key BBB features, endothelial cell markers, and activity for the transferrin receptor-mediated transcytosis, essential in the maintenance of the BBB role. The brain microvascular endothelium revealed few molecular differences from distinct regions, suggesting a closely homogeneous function of the BBB across the CNS. The present characterization of the NHP-derived BBB constitutes a potent tool for prediction of the BBB permeability in vivo to biologic compounds, and for the identification of new brain-specific mechanisms for improved drug delivery.

## 2. Materials and Methods

### 2.1. Animals

Male and Female Cynomolgus monkeys (Macaca fascicularis) aged from 4.8 to 5.9 years were purchased from Le Tamarinier and Noveprim Ltd. (Mahebourg, Mauritius). Six animals were group-housed in aviaries or interconnected mobile cages and two animals were individually housed in interconnected mobile cages. Animals were housed under controlled conditions (20–24 °C, 40–70% humidity, 10–15 renewals per hour of filtered, non-recycled air, 12-h light cycle) with free access to filtered tap water and daily distribution of expanded diet (SDS, France) and fruits or vegetables. Animals used for the isolation of brain microvessels were at disposal following pre-clinical studies. Prior to the isolation of brain cortical microvessels, animals were submitted to a washout period of a minimum of 1 month. All animal experimental protocols were reviewed and approved by the Ethics Committee for the Protection of Laboratory Animals (CEPAL #21) of Sanofi, Vitry-Alfortville Research Center, France, and are in compliance with the ARRIVE guidelines

### 2.2. Isolation of Brain Microvessels from Cynomolgus Monkey Cortex, Brainstem, Cerebellum, Hippocampus and Striatum

**Figure 1:**
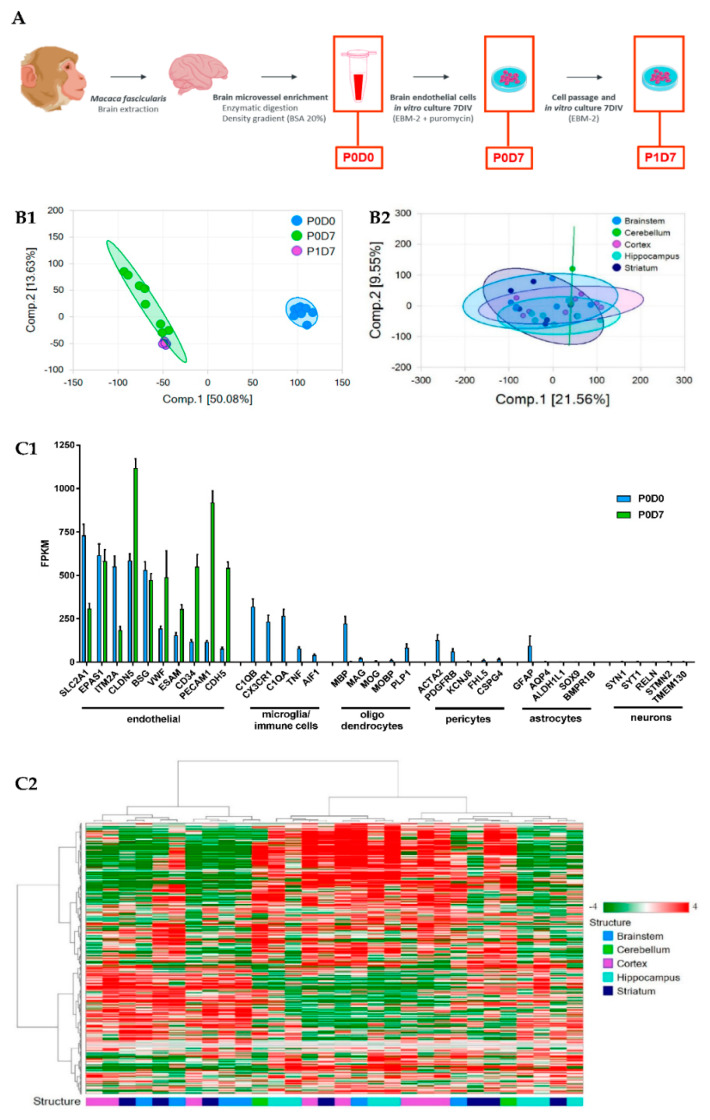
Brain endothelium transcriptome of samples prepared from non-human primates (NHP). (**A**) Diagram of the procedure conducted for the isolation of NHP-derived brain microvessels and in vitro selection of brain endothelial cells (BECs). (**B1**) Principal Component Analysis (PCA) of brain cortical microvascular samples clustered in 3 groups (P0D0 *n* = 8, P0D7 *n* = 8, P1D7 *n* = 6). (**B2**) PCA of brain endothelial samples clustered in 5 groups (cortex *n* = 8, brainstem *n* = 6, hippocampus *n* = 7, striatum *n* = 7, cerebellum *n* = 2). (**C1**) Comparison of the expression levels of endothelial cell and non-endothelial cell markers in the brain microvascular P0D0 and P0D7 fractions. An expression threshold of one FPKM in at least half of the independent replicates (P0D0, *n* = 8; P0D7, *n* = 8; P1D7, *n* = 6; FPKM ≥ 1 in *n* ≥ 4 samples) was applied in order to consider only the differentially expressed gene having a significant expression level. Gene levels of brain endothelial cell markers are enriched in P0D0 and P0D7 samples, and even though RNA levels of markers for microglia (or immune cells), oligodendrocytes, pericytes, astrocytes and neurons can be detected in P0D0 samples, their expression is off in P0D7. Results represent average FPKM and are expressed and mean ± SEM. (**C2**) Hierarchical clustering of BECs from the selected brain structures showing a lack of clustering per brain structure. An expression threshold of one FPKM in at least 3 samples was applied to ECs derived from different regions (cortex *n* = 8, brainstem *n* = 6, hippocampus *n* = 7, striatum *n* = 7, cerebellum *n* = 2) for the analysis of differentially expressed genes.

Brains from Cynomolgus monkeys were collected shortly after the euthanasia of the animal into ice-cold Hibernate A medium (ThermoFisher, Illkirch-Graffenstaden, France). All subsequent steps were performed at 4 °C and under a biological safety cabinet. Brain cortex, brainstem, cerebellum, hippocampus, and striatum structures were isolated, and the subsequent brain tissue placed in petri dishes containing ice-cold HBSS. The meninges and the cortical white matter were removed. The collected tissues were transferred into a new sterile container with HBSS, finely minced with a scalpel, and then pelleted by centrifugation (5 min at 600× *g*, 4 °C). The pellet was resuspended in a collagenase/dispase^®^ solution (Roche, Meylan, France, Collagenase 0.1 U/mL; Dispase 0.8 U/mL prepared in Ca^2+^/Mg^2+^ free HBSS) containing type I DNAse at 20 U/mL and TLCK at 0.147 μg/mL, and incubated at 37 °C for 60 min, under vigorous agitation. The digested tissue was carefully homogenized, and centrifuged for 5 min at 600× *g*, 4 °C. The resultant pellet was resuspended in HBSS containing 20% BSA and centrifuged at for 30 min at 2000× *g*, 4 °C. The myelin ring-containing supernatants were discarded, and the vessel-containing pellet was resuspended and re-incubated in the collagenase/dispase^®^ solution in presence of DNAse and TLCK for 30 min at 37 °C. This suspension was re-pelleted by centrifugation (5 min at 600× *g*, 4 °C), and the final pellet (named P0D0 fraction from this point onwards) is resuspended in endothelial cell medium (EBM-2 supplemented with Kit EGM-2 MV SingleQuots, Lonza, Basel, Switzerland) containing 3 µg/mL puromycin, before set up in pre-coated (collagen IV 100 µg/mL, fibronectin 10 µg/mL, Sigma, Saint Quentin Fallavier, France) cell culture flasks, and incubated at 37 °C, 5% CO_2_ for 7 days. Every 2 days the cell medium was changed and the supplemented puromycin concentration lowered to 2 µg/mL, and subsequently removed. Following 7 days of expansion, BECs from cortex, but not from the other 4 brain structures (named P0D7 fraction from here onwards) were further singularized and re-plated de novo for further 7-day cell expansion (P1D7) (Figure 1A).

### 2.3. Transcriptome Sequencing (RNAseq) and Expression Analysis

#### 2.3.1. RNA Samples for Library Preparation

Frozen cell pellets from the cellular P0D0 (cortex), P0D7 (cortex, brainstem, cerebellum, hippocampus, striatum) and P1D7 (cortex) fractions were lysed using QIAzol Lysis Reagent (Qiagen, #79306, Courtaboeuf, France). Total RNA was then isolated from the lysates on a QIAcube instrument (Qiagen) using the RNeasy Mini QIAcube kit (Qiagen, #74116) and following the manufacturer’s instructions. The RNA concentration was determined using the Qubit RNA HS Assay Kit (Invitrogen, #Q32852, Illkirch-Graffenstaden, France) and the quality and integrity (RIN) was assessed on a Bioanalyzer 2100 (Agilent Technology, Les Ulis, France) with an Agilent RNA 6000 nano kit (Agilent Technology, #5067-1511).

#### 2.3.2. RNA Sequencing (RNAseq)

The RNAseq libraries were prepared with 30 ng of input total RNA using the NEBNext Ultra II Directional RNA Library Prep Kit for Illumina (New England Biolabs, #E-7765S, Évry-Courcouronnes, France) with the NEBNext rRNA Depletion Kit (New England Biolabs, #E6310L) and following the manufacturer’s instructions. The libraries were then paired end sequenced (75 cyclesx2) on the NovaSeq 6000 instrument (Illumina, Paris, France) using the NovaSeq 6000 SP Reagent Kit (300 cycles; #20027465, Illumina).

RNA-seq data analysis was performed using ArrayStudio (Qiagen, Courtaboeuf, France). Briefly, raw data QC is performed then a filtering step is applied to remove reads corresponding to rRNAs as well as reads having low quality score or shorter than 25 nt. Reads were further mapped to the Cyno.WashU 2013 genome, based on the contigs assembled from a WGS project submitted by Washington University (WashU) in 2013, using OSA4 [12] (Omicsoft Sequence Aligner, version 4, Qiagen) and quantified using Ensembl.R94 model of transcriptome, paired reads were counted at gene level. Differential analysis of gene expression was performed at gene level using DESeq2 [13]. The variable multiplicity was taken into account and false discovery rate (FDR) adjusted p-values calculated with the Benjamini-Hochberg (BH) correction [14]. Functional analysis of differentially expressed genes was performed using IPA (Qiagen) [15]. Gene Set Enrichment analysis was performed using Ingenuity and GSEA software [16,17]. High-throughput sequence data are available on the Gene Expression Omnibus (GEO) under the GSE154901 accession number. Read depth from RNAseq was counted as fragments per kilobase per million mapped fragments (FPKM). FPKM read counts were obtained for each BEC fraction (P0D0, P0D7, P1D7). A minimum of 1 FKPM in at least half of the observations per group, and in at least one of the groups, was required for a gene to be considered as expressed and included in the analysis.

### 2.4. Co-Culture Experiments

NHP-derived BECs were seeded onto 12-well plate transwells (Corning Transwell polycarbonate filters, 0.4 μm pore diameter; 1.12 cm^2^, Corning, Sigma, Saint Quentin Fallavier, France), previously coated with collagen IV/fibronectin (Sigma, Saint Quentin Fallavier, France) at a density of 60,000 cells/well in supplemented EBM-2 medium. Immediately following plating, BEC-coated transwells were then placed into plates with either no other cell type (monoculture), or with rat primary astrocytes (80,000 cells/well) plated onto the bottom of the plate 4 days before, in MEM-α/F-12 cell medium (1:1), 10% FBS, 1% PSN and 5 ng/mL bFGF. All transcytosis experiments occurred immediately following 4 days or 7 days in monoculture or co-culture.

### 2.5. Resistance Measurements

Trans-endothelial electrical resistance (TEER) was measured every 24 h following the sub-culture of BMECs onto transwell filters. Resistance was recorded using an EVOM2 epithelial voltohmmeter coupled to a cell culture cup chamber (ENDOHM-12G) (World Precision Instruments, Hitchin, Hertfordshire, United Kingdom). TEER values were presented as Ω × cm^2^ following the subtraction of an un-seeded Transwell and multiplication by 1.12 cm^2^ to account for the surface area. TEER measurements were taken three independent times on each sample and at least in triplicate filters for each experimental condition.

### 2.6. In Vitro Transcytosis and Permeability Measurements

Test (2 µg/mL human holo-Transferrin or 1 µg/mL of internally developed anti-human/cynomolgus TFRC antibody) and control molecules (10 µg/mL of FITC-coupled 70 kDa Dextran or 1 µg/mL mouse IgG, clone MG1-45, BioLegend) were added onto the upper chamber on day 4 (96 h following NHP-derived BMEC seeding on transwell). Fresh EC medium with none of these compounds was added onto the bottom chamber. Final aliquots from both chambers were taken 240 min following incubation at 37 °C, 5% CO_2_. Compound levels in mother solutions (*T* = 0 min), upper and lower compartments (*T* = 240 min) were determined by electrochemiluminescent immunoassay (MESOQuickPlex SQ120, MesoScale Discovery, Rockville, MD, USA) and by fluorescence measurement (485 nm excitation and 530 nm emission, using a multiwell-plate reader). Permeability coefficients were calculated based on the cleared volume of Dextran or holo-Transferrin from the top chamber to the bottom chamber using the following formula:

(1) $$Papp\;\left(\mathrm{cm}/\min\right)\;=\left(\frac{V}{A\;.\;Cluminal}\right).\left(\frac{Cabluminal}{t}\right)$$

where *V* = volume of cell medium in the bottom chamber (mL), *A* = surface area of the insert (cm^2^), *C*_luminal_ = compound concentration loaded in the upper chamber (µM), *C*_abluminal_ = compound concentration measured in the bottom chamber (µM); *t* = time of the assay (min).

### 2.7. Western Blot

NHP-derived BECs were lysed using ice-cold RIPA buffer (CST) containing protease inhibitor cocktail (ThermoFisher). Lysates were sonicated for 5 min, centrifuged at 15,000× *g* for 15 min, and supernatant fractions were collected. These were loaded into 4–12% Tris-Glycine SDS-page gels (Invitrogen, Illkirch-Graffenstaden, France), and let to migrate for 1h at 180V. Samples were then transferred onto PVDF membranes using an iBlot™ 2 Dry Blotting System (Invitrogen) on the P0 program (20 V for 1 min, 23 V for 4 min, 25 V for 2 min). PVDF membranes were then rinsed with Tris-buffered saline with 0.1% Tween 20 (TBST) and blocked for 1 h in 5% non-fat dry milk in TBST (blocking buffer). Membranes were first probed overnight at 4 °C with primary antibodies in blocking buffer, and then probed with secondary antibodies diluted in TBST for 1 h at room temperature (1:10,000 diluted HRP-coupled goat anti-mouse IgG or goat anti-rabbit IgG, GE Healthcare). Following secondary antibody incubation, membranes were rinsed thoroughly with TBST, imaged using a LICOR Odyssey Imager and bands quantified using Multi-Gauge v3.0.

### 2.8. Immunocytochemistry of Tight Junctions

Immunocytochemistry was conducted on NHP-derived BECs following mono-culture conditions. Cultured cells were fixed in 4% p-formaldehyde for 15 min, at room temperature, and subsequently permeabilized and blocked in Blocking Buffer Odyssey LiCor containing 0.2% Triton X-100. Primary antibodies were incubated overnight (Anti-Claudin 5 Monoclonal (4C3C2) 1:200 #35-2500; Anti-ZO-1 Polyclonal, 1:200 #61-7300; Anti-Occludin Monoclonal (OC-3F10), 1:100 #33-1500, ThermoFisher; Anti-VE Cadherin Polyclonal, 1:500 #ab33168, Abcam, Amsterdam, The Netherlands), and appropriate secondary antibodies conjugated with Alexa fluorophores (Invitrogen) and Hoechst 33432 (Invitrogen) for nuclei staining were subsequently used. Images were acquired on a Perkin Elmer Operetta CLS™ system.

### 2.9. Statistical Analysis

The statistical significance of differences between groups was analyzed using GraphPad Prism v7.0 software (GraphPad Software, San Diego, CA, USA) *t*-test (unpaired, parametric, equal standard deviation, two-tailed), Mann–Whitney *t*-test (unpaired, nonparametric, two-tailed) and Friedman test (matched data, nonparametric) with post hoc Dunn’s multiple comparison test. The application of these statistical methods to specific experiments is noted in the figure legends.

## 3. Results

### 3.1. Transcriptional Profiling of Brain Microvasculature

For the detection of differentially expressed genes, we performed statistical analysis with DESeq2 [13]. Transcriptional profiles of the different cortical EC populations were compared to one another, while ECs from different brain regions were compared to those obtained from brain cortex, always using a threshold of 1.5-fold difference in the expression and a BH-adjusted *p*-value (*p*_adj_ ≤ 0.05).

In the principal components analysis (PCA), a clustering of independent replicates is seen for each fraction, supporting a low variability between independent samples (Figure 1(B1)). The PCA of the different endothelium transcriptomes also evidences that brain ECs set in culture (P0D7, P1D7) have molecular signatures that are relatively distinct from the freshly obtained BEC-enriched fraction P0D0 (Figure 1(B1)). Such difference of P0D7 and P1D7 towards the P0D0 fraction is partially due to the presence of RNA from cell types other than endothelial cells in the P0D0 fraction. In fact, the enzymatic digestion and gradient centrifugation procedure used for obtention of brain microvessels (P0D0) yields a highly enriched fraction yet presents RNA of pericyte, glial, neuronal, and circulating blood cell origin. This also explains the important set of genes seeing their expression downregulated in P0D7 vs. P0D0 (5089 out of a 13,485 gene population, see Figure S1). On the other hand, an up-regulation of 3884 out of 13,485 genes is seen from P0D0 to P0D7 (≥1.5-fold, *p* ≤ 0.05), most probably representing endothelial-specific genes, and thus reflecting the in vitro selection of the endothelial cell population. The degree of purity of P0D0 and in vitro-selected P0D7 and P1D7 samples was assessed through probing the transcriptome data for the expression of 5 acknowledged cell type specific genes [18,19,20,21] for astrocytes (*BMPR1B*, *GFAP, ALDH1L1, SOX9, AQP4*), neurons (*TMEM130*, *RELN*, *STMN2, SYT1, SYN1*), oligodendrocytes (*MAG*, *PLP1, MOG, MOBP, MBP*), and microglia (*AIF1*, *C1QA, C1QB, CX3CR1, TNF*), and 10 genes for endothelial cells (*SLC2A1/GLUT1*, *EPAS1*, *ITM2A*, *CLDN5*, *BSG*, *CD34*, *VWF*, *PECAM1*, *CDH5*, *ESAM*) (Figure 1(B2)). All non-endothelial cell markers see their expression being switched-off in P0D7 and P1D7 cell populations, while the expression of the selected endothelial genes is either maintained or even increased in both the P0D7 and P1D7 cell populations, in comparison to P0D0 (Figure 1(C1)).

All BEC samples from five brain regions (brainstem, cerebellum, cortex, hippocampus, striatum) qualified according to the raw data quality control analysis, with the exception of 2 cerebellum with low RNA levels (consequently excluded from the subsequent analyses). The PCA analysis and hierarchical clustering suggest low variability between independent samples (Figure 1(B2)), and similar molecular signatures of endothelium transcriptomes from different brain structures as no distinct clustering of samples belonging to the same structure is formed (Figure 1(C2)). Accordingly, only 173 genes were found to be differentially expressed (fold-change > ±1.5, *p* < 0.05) in BECs from other brain structures vs. those from brain cortex (Figure S2).

### 3.2. Endothelial Junction Mediators: Expression Levels and Transcriptional Changes

**Figure 2:**
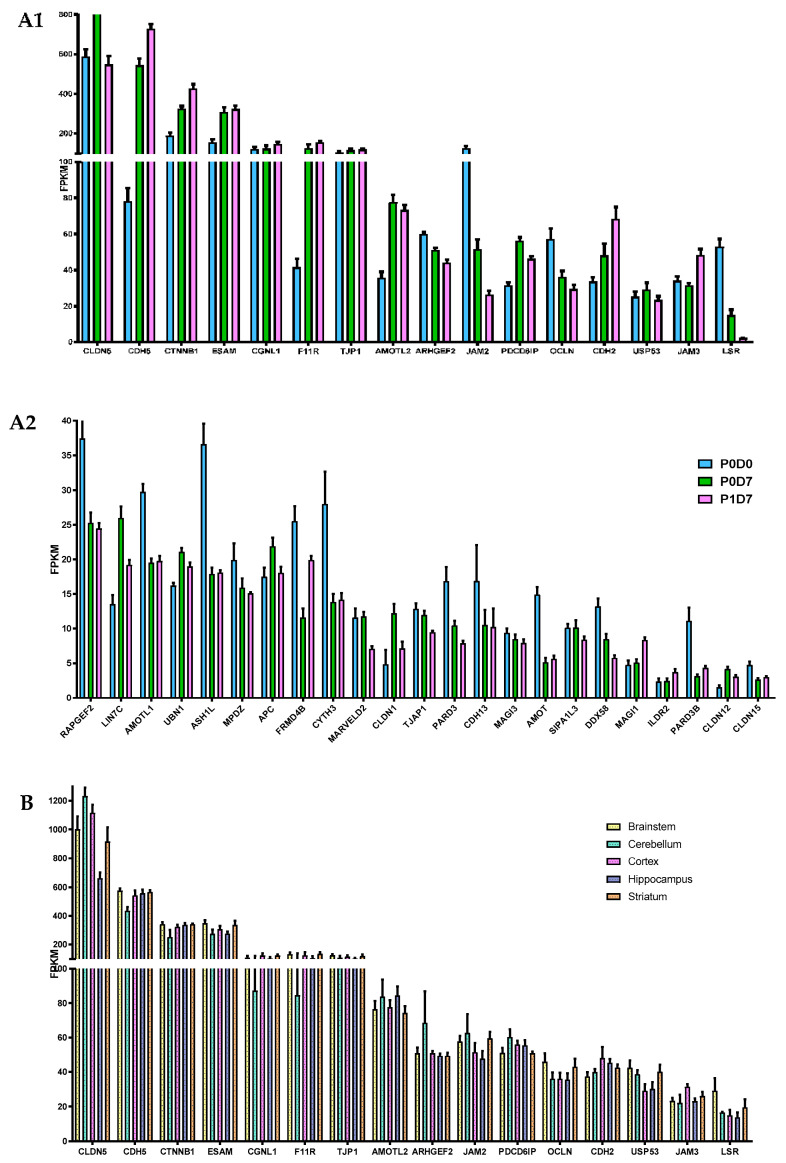
Comparison of the expression levels of several molecular participants in the formation of tight junctions at the brain microvasculature. (**A**) Gene levels of tight junction elements are globally maintained across P0D0, P0D7 and P1D7 fractions, where (**A1**) represents the most expressed genes associated with tight junction formation and regulation, and (**A2**) genes showing lower average FPKM values. Results are expressed as average FPKM and are expressed and mean ± SEM (*n* = 6–8). (**B**) Comparison of the expression levels of several molecular participants in the formation of tight junctions in brain microvascular endothelial cells from 5 different brain regions. Results are expressed as average FPKM and are expressed and mean ± SEM (*n* = 2–8).

The present RNAseq analysis evidences *CLDN5* as the major claudin transcript present in the cynomolgus brain vasculature, but *CLDN1*, *CLDN12,* and *CLDN15* expression at the 3 different in vitro stages is also detected, at considerably lower levels (Figure 2A). *CLDN3* and *CLDN6*, previously reported at the mouse BBB [22,23] were not detected. Other key participants in the formation of tight junctions at the BBB, in particular JAMs (*F11R*, *JAM3*), *TJP1* (ZO-1), and cadherins CDH5, CDH2, and ESAM seem to maintain high levels of expression in isolated BECs, even after sub-culturing in vitro (Figure 2A). JAM2 and to a lesser extent OCLN are progressively downregulated from P0D0 to P1D7, but present nonetheless relevant levels of expression. The expression of *CTNNB1* (β-catenin), regulator of CNS-specific angiogenesis and of the expression of tight junctions at the BBB [23,24,25,26], is dominant over the three endothelial fractions, along with other genes associated to tight junction correct assembly and maintenance, such as *USP53*, *PDCD6IP*, *ARHGEF2*, *MPDZ,* and *AMOTL1-2* (Figure 2A), further sustaining the robustness of the NHP-derived BBB model. Lipolysis stimulated receptor (*LSR*), although downregulated (3.5-fold, *p* < 0.001, P0D7 vs. P0D0)*,* together with stable levels of *CGNL1 MARVELD2* found in vitro, which are known to participate in the formation of epithelial and endothelial junctions, and enriched in mouse BECs versus periphery [27,28] were also identified in our analysis.

Most major participants in the regulation and formation of cellular junctions, such as *CDH5*, *TJP1,* or *OCLN*, did not see their expression levels differ between endothelial cells obtained from the brain microvasculature of the five analyzed regions, except for increased transcript levels of *CLDN5* and *CLDN1* found in BECs from the brainstem and cortex vs. hippocampus (*p* < 0.05).

### 3.3. Transporter Systems at the BBB: Expression Levels and Transcriptional Changes

#### 3.3.1. Receptor-Mediated Transcytosis

**Figure 3:**
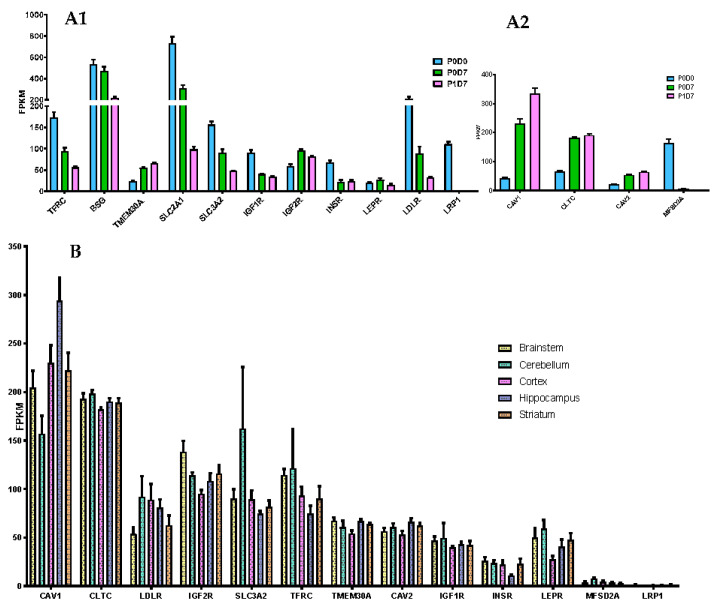
Expression of receptors capable of mediating transcytosis. (**A**) Comparison of the expression levels (FPKM) of selected receptors (**A1**) and key internalization pathway genes (**A2**) present in the brain microvascular P0D0, P0D7 and P1D7 fractions. Results represent average FPKM and are expressed and mean ± SD (*n* = 6–8). (**B**) Comparison of the expression levels (FPKM) of selected receptors and key internalization pathway genes present in the brain microvascular endothelial cells from different brain regions. Results represent average FPKM and are expressed and mean ± SEM (*n* = 2–8).

RMT constitutes a reference strategy to overcome the BBB and promote efficient and specific delivery into the CNS of therapeutic agents. TFRC, highly expressed and enriched in the brain microvasculature [29,30], and the only validated RMT-mechanism evidencing an enhanced brain exposure of biotherapeutics [31,32,33,34,35], sees its expression levels being downregulated from P0D0 to P1D7, but retaining substantial expression levels in vitro (Figure 3A). Insulin receptor (INSR) [36,37,38,39], LRP1 [37,40,41,42], IGF1R [43], LDLR [44], basigin (BSG), and SLC2A1/GLUT1 [37] have been targeted using antibodies and/or peptides as attempts to improve brain uptake, but such studies did not generate much encouraging results. Shuttles targeting novel receptors have also been described, such as SLC3A2/CD98hc [37] and TMEM30 [45,46], yet poorly characterized. A sustained relevant expression from P0D0 to P1D7 is seen for the transcript levels of most of these receptors, except for *LRP1*, which is considerably suppressed in vitro (Figure 3(A1)). The absent expression of *LRP1* is coherent with previous findings revealing strong LRP1 expression levels found in pericytes [47,48] but not in selected brain endothelial cells [47,49]. *TMEM30A* and *IGF2R* levels see a relative increase in vitro, likely due to the endothelial cell selection in culture. Expression levels of *TFRC* did not differ between endothelial cells from the different analyzed brain structures (Figure 3B). Increased *IGF2R* and *TMEM30A* transcript levels were found in the brainstem-derived endothelium vs. cortex-derived endothelium (*p* < 0.05), while *INSR* showed to be significantly inferior in hippocampus vs. brainstem (≥1.5-fold change, *p* < 0.05, vs. striatum and cortex (≥1.5-fold change, *p* > 0.05).

Clathrin [36,50,51,52] and caveolin-mediated endocytosis [53,54,55,56] constitute the major pathways of internalization in RMT. The expression of clathrin (*CLTC*), caveolin-1 and *2* (*CAV1*, *CAV2*) found in P0D0 brain microvessels is up-regulated and preserved at P0D7 and P1D7 timepoints (Figure 3(A2)). Transcript levels of *MFSD2A* are downregulated in vitro, suggesting a suppression of the negative regulation of caveolae formation, which may consequently be exacerbated in this context (Figure S3). No gene implicated in RMT internalization pathways registered a differential expression pattern in BECs obtained from the different five brain regions.

#### 3.3.2. Carrier Transporters

##### Comparison of Cortical Brain Endothelial P0D0, P0D7 and P1D7 Fractions

The presence of cell membrane transporters at the BBB allows a perfect control of the flux of molecules between the blood and the brain, either by efflux ATP-binding cassette (ABC) transporters (e.g., ABCB1/MDR1/P-glycoprotein) [57], or highly specific solute carriers (SLC) (e.g., SLC2A1/GLUT1) [58]. Expression trends of such transporters were therefore Investig.igated across the three studied cell fractions. Based on the overall samples, we detected a total of 398 transporters, 249 of these detectable at ≥1 FPKM in at least half of the samples from at least one of the analyzed groups.

**Figure 4:**
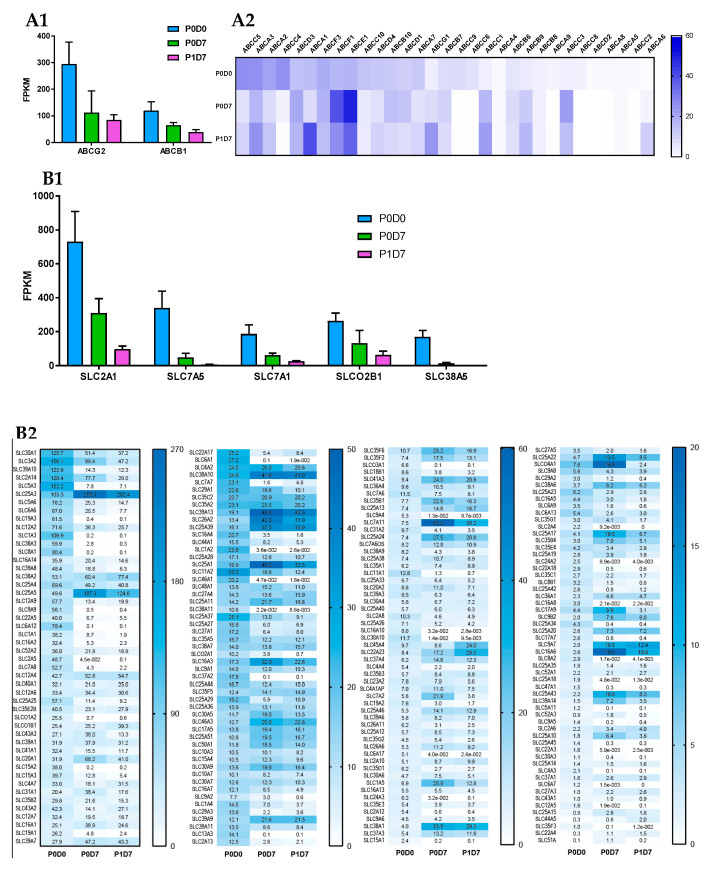
Changes in mRNA levels of ABC and SLC transporters across P0D0, P0D7 and BEC fractions. (**A**) Comparison of the expression levels (FPKM) between the different ABC transporters present in the brain microvascular P0D0, P0D7 and P1D7 fractions. (**A1**) ABCB1 and ABCG2 are by far the most expressed ABC transporters across the 3 cell population fractions. Results represent average FPKM and are expressed and mean ± SD (*n* = 6–8). (**B**) Comparison of the expression levels (FPKM) between the different SLC transporters present in the brain microvascular P0D0, P0D7 and P1D7 fractions. (**B1**) SLC2A1, SLC7A5, SLC7A1, SLCO2B1, SLC3A2 and SLC38A5 are the most expressed SLC transporters at P0D0 but are downregulated in vitro. Results represent average FPKM and are expressed and mean ± SD (*n* = 6–8).

ABC transporters: Known major drug efflux transporters, like ABCG2 (BCRP) and ABCB1, presented the highest expression levels among the 33 ABC transporters found in the three studied endothelial cell populations. *ABCG2* and *ABCB1* transporters were downregulated in vitro, but nonetheless remained the most expressed ABC transporters in the P0D7 and P1D7 sampling groups (Figure 4A). *ABCC4* expression levels showed to be third highest among ABC transporters in the P0D0 fraction, but its expression is considerably depleted in vitro. Similarly, *ABCC6* and *ABCC9* were detected in freshly isolated brain microvessels, followed by downregulation in vitro. *ABCC9*, but not *ABCC6*, have previously been reported to be present at the BBB microvasculature [59,60], but it likely originates from pericytes [61]. On the other hand, expression of *ABCC1* is considerably upregulated from P0D0 to P0D7 and P1D7 culture conditions (despite controversial prior reports [62,63,64]), and similar trends are also observed for *ABCB9* (reported in rat brain microvessels [60], *ABCC3* and the lipid transporter *ABCG1* (reported in porcine brain endothelium [65]). The upregulation of these 5 ABC transporters versus P0D0 is most likely due to the positive selection of endothelial cells in culture, and not a cell derivation in vitro, given that these very same five ABC transporters showed to be positively correlated with the trend of *PECAM1* expression across the three endothelial cell fractions (Pearson correlation, *p* < 0.001, Figure S5). Other transporters which showed considerable expression at P0D0 and for which their expression was relatively maintained in vitro are the cholesterol efflux transporters *ABCA1*, *ABCA2* (reported in human brain microvessels [66,67]) and *ABCA3*, the multidrug transporter *ABCC5*, but also those of *ABCD3* [60], *ABCE1* (expression associated with cell growth and apoptosis inhibition) and *ABCF1* (Figure 4A), for which the eventual played role at the BBB is absolutely unknown. A global heatmap plot representation of a hierarchical clustering of the FPKM variability of ABC transporters expressed genes across fractions is available in Figure S4.

SLC transporters: The SLC superfamily comprises known transporters of energy metabolites (e.g., SLC2A, SLC16A families), amino acids and neurotransmitters (e.g., SLC1A, SLC7A families), ions (e.g., folate/thiamine transporter family SLC19A, zinc transporter family SLC39A), organic anions (SLCO family), organic cations (SLC22A family), and others. Transporters such as SLC2A1 (GLUT1) [68,69], SLC7A5 (LAT1) [70,71], SLC7A1 (CAT1) [72], or SLCO2B1 (OATP2B1) [73,74] are known to participate in mediated-transport at the BBB. The analysis of the transcriptomic data indicates that the glucose transporter *SLC2A1*, the amino acid transporters *SLC7A5*, *SLC7A1* and *SLC3A2* (the latter known to form heterodimers with SLC7 family members, e.g., with SLC7A5 to form the neutral large amino acid transporter LAT-1), *SLCO2B1*, but also *SLC38A5* [75,76] (SNAT5, carrier for glycine, alanine, serine, glutamine, asparagine, and histidine) are the most abundantly expressed SLC transporters in P0D0 (Figure 4B). Their expression was downregulated in P0D7 and P1D7 endothelial fraction yet maintaining relevant levels of expression as they remained the most expressed SLCs. *SLC7A5* and *SLC38A5* although known to be prominently expressed at the BBB [76,77,78] represented important exceptions as their expression dramatically dropped from an average 339 or 169 FPKM, respectively, at P0D0 to approximately 6 and 2 FPKM, respectively, at P1D7. As SLC7A5 heterodimerizes with SLC3A2 to function as a sodium-independent, high-affinity transporter mediating uptake of large neutral amino acids such as phenylalanine, tyrosine, L-DOPA, leucine, histidine, methionine and tryptophan, its modest expression might limit the formation of such complexes and partially compromise LAT-1 activity. Expression of *SLC7A11* (xCT, cystine/glutamate antiporter, also forming dimers with SLC3A2) is considerably upregulated in vitro, finding that has already been described [75], and which is associated with increased oxidative stress conditions [79]. Given the similar transcript levels of SLC3A2 and SLC7A11 found at P0D7-P1D7, formation of this heterodimer in vitro should be sustained.

Some SLC38A transport family members (Na^+^-dependent transport of neutral amino acids), namely *SLC38A1* (transport of glutamine), *SLC38A2*, and *SLC38A10* (proton antiporter, orphan transporter) are mainly upregulated in vitro, and account for high levels of expression among SLCs, notably *SLC38A2* (Figure 4B). Other simple sugar transporters from the SLC2A family were found to be significantly expressed in the cynomolgus brain microvascular enriched fraction, such as *SLC2A5* (corroborated by [80]) and *SLC2A13-14*. Although the expression of *SLC2A5* was also depleted in vitro, an important expression of both *SLC2A10* and *SLC2A14* was found at both P0D7 and P1D7. The expression levels of some SLC12A (cation-coupled chloride transporters)—*SLC12A2*, *SLC12A4*, *SLC12A6*—and SLC16A (proton-coupled monocarboxylate uptake, such as lactate and pyruvate, but also drugs, including statins) members—*SLC16A1*, *SLC16A3*, *SLC16A14*—were also among the most relevant SLC transporter expressed at the cynomolgus BBB, coherent with previous reports [81,82], and whose levels of expression were maintained in vitro. On the other hand, even though a broad expression level was found at P0D0 for *SLC16A2*, known to carry cellular import of thyroid hormones, its expression was substantially downregulated across both P0D7 and P1D7 fractions. High levels of expression of SLC25A transporter family members (mitochondrial carriers, e.g., exchange of ADP/ATP, citrate, phosphate), namely *SLC25A1*, *SLC25A3*, *SLC25A4*, *SLC25A5*, were also observed from P0D0 to P0D7/P1D7 conditions (Figure 4B). They account for nine out of 25 SLC transporter expression positively correlated with the expression of PECAM1 across the three endothelial cell populations (Figure S5). Other SLC transporters found to be present across the three endothelial cell fractions are the zinc efflux family SLC30A (*SLC30A1*, *SLC30A9*) and the zinc uptake family SLC39A (*SLC39A1*, *SLC39A7*, *SLC39A9*, and *SLC39A13*), suggesting the importance of the fine tuning of the zinc intracellular concentration levels for the cellular homeostasis and proper functioning. Our present findings allow to better establish a set of BBB highly expressed transporters which may provide novel insights into its role for the maintenance of the CNS homeostasis and nutrient requirements, but also for identifying novel molecules to target to enhance drug delivery into the CNS. A global heatmap plot representation of a hierarchical clustering of the FPKM variability of SLC transporters expressed genes across the 3 fractions can be consulted in the (Figure S4).

##### Comparison of Brain Microvascular Endothelial Cells from 5 Brain Regions at P0D7

**Figure 5:**
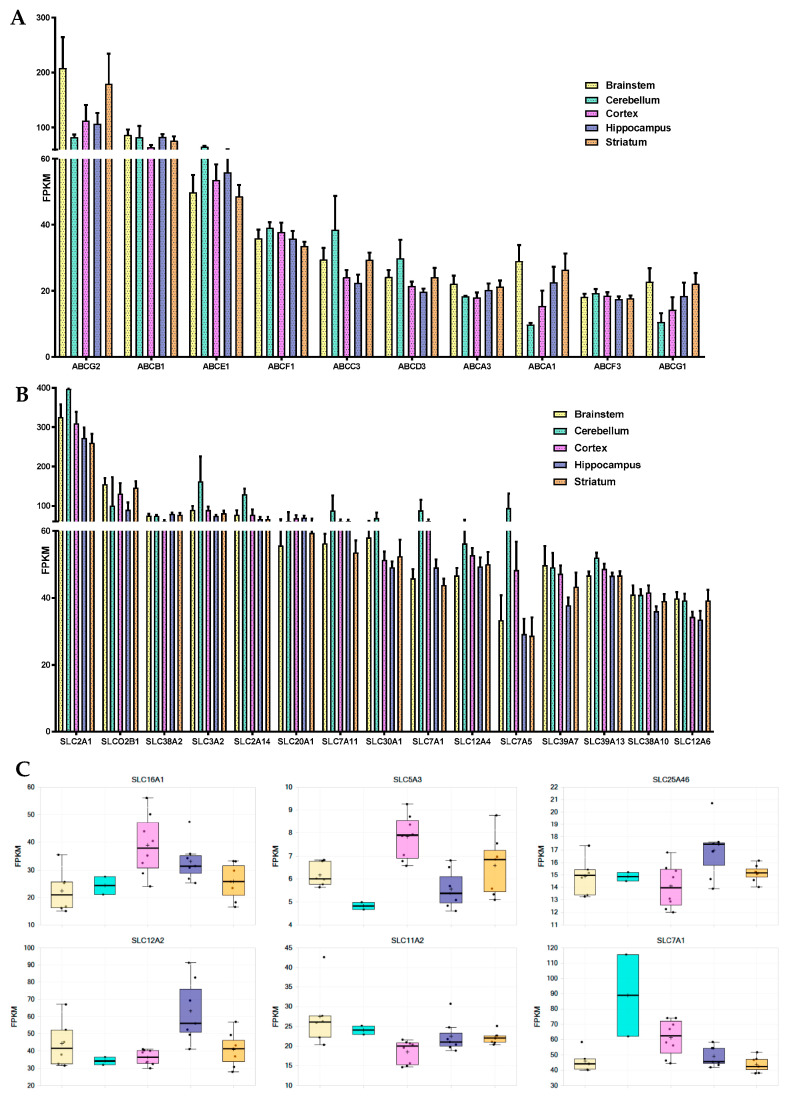
Differences in the mRNA levels of ABC and SLC transporters in the brain endothelium from brainstem, cerebellum, cortex, hippocampus and striatum. (**A**) Representation of the 10 ABC transporters with highest transcript levels in BECs from across the 5 studies brain regions. Expression levels are represented as average FPKM ± SEM. (**B**) Representation of the 15 SLC transporters (typically at plasmatic membrane, excludes SLC25A family, mitochondrial transporters) with highest transcript levels in BECs from across the 5 studies brain regions. Expression levels are represented as average FPKM ± SEM. (**C**) Representation of the 6 SLC transporters for which there is a differential expression (*p* < 0.05) in BECs cultivated from a given brain structure in comparison to another brain region.

No ABC transporter genes were found to be differentially expressed in the brain endothelium from either brainstem, cerebellum, hippocampus, or striatum in comparison to that from brain cortex. As in the cortical-derived brain endothelium, *ABCG2*, *ABCB1,* and *ABCE1* were the most expressed ABC transporters at the BBB from the analyzed four brain regions (Figure 5A). *ABCG2* levels displayed a tendency to be higher in the brainstem and striatum when opposed to those from cortex and hippocampus. *ABCA6* expression levels also showed trendily increased in hippocampus-derived endothelial cells versus brain cortex, although with no statistical difference (≥1.5-fold change, *p* > 0.05).

The 16 SLC transporters found to be differentially expressed across the endothelium from the 5 brain structures are represented in Figure 5C. Among the SLC transporters with the highest expression levels across the five studied structures are *SLC2A1, SLCO2B1, SLC38A2, SLC3A2, SLC2A14,* and *SLC20A1* (Figure 5B). Endothelial cells derived from the hippocampal BBB were shown to be richer in *SLC12A2* and *SLC25A46* in comparison to cortex, but poorer in *SLC5A3* (*p* < 0.05). Levels of expression of *SLC7A1* and *SLC16A1* were found to be lower in BECs from both brainstem and striatum in comparison to brain cortex, while levels of SLC11A2 were shown to be higher in brainstem vs. brain cortex endothelial cells (*p* < 0.05).

Cerebellum-derived brain endothelial cell transcript levels of *SLC3A2*, *SLC1A5*, *SLC7A1*, *SLC7A5*, *SLC6A9*, and *SLCO4A1*, although with no statistical value given its size sample (*n* = 2), showed to be increased than those found in the brainstem, striatum, and hippocampal BBB. The expression of other major SLC transporters in BECs, such as *SLC7A11*, *SLC30A1,* or *SLC12A4*, did not seem to differ between the five studies brain structures.

### 3.4. Function of Transferrin Receptor-Mediated Transcytosis

**Figure 6:**
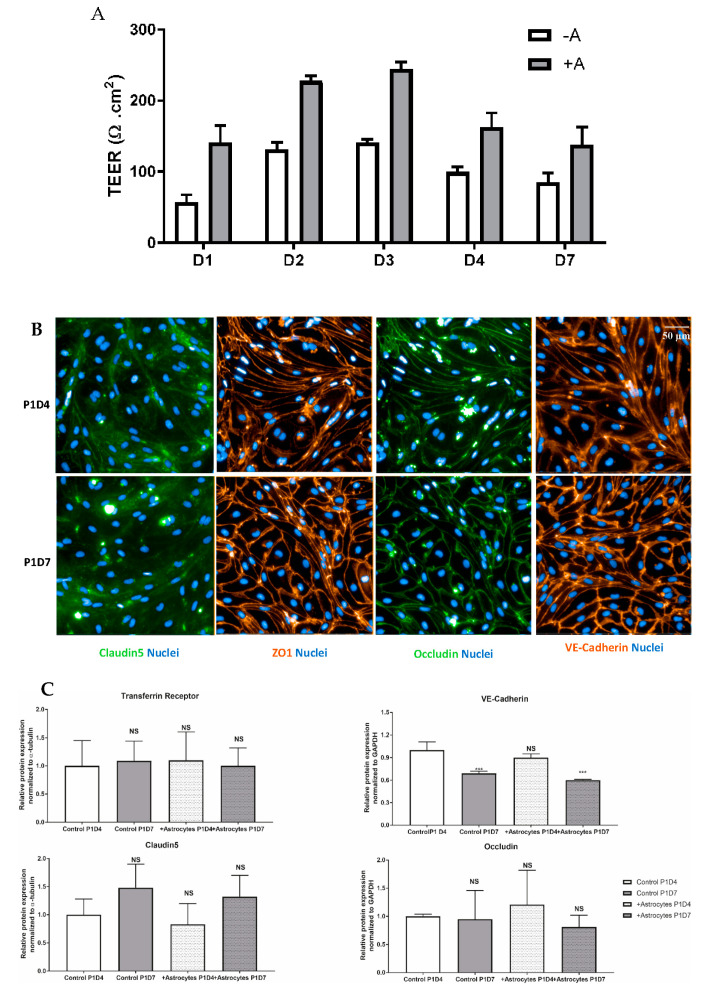
Profiling of essential proteins for maintenance of a BBB phenotype and transport properties in NHP-derived BECs. (**A**) TEER values registered across endothelial cells following several days in monoculture (−A) or in co-culture with astrocytes (+A). Data are presented as mean values from 3 independent experiments, each containing 4–8 filter inserts. (**B**) Immunofluorescence staining of several cell-cell junction proteins in BECs. Protein staining was obtained in the NHP-derived BBB model at P1 following either 4 or 7 days in vitro (P1D4 and P1D7, respectively) in the absence of primary rat astrocytes. (**C**) Relative protein expression of cell-cell junction and TFRC proteins in BECs. Relative protein levels were obtained in the NHP-derived BBB model at P1 following either 4 or 7 days in vitro (P1D4 and P1D7, respectively) in the absence or presence of primary rat astrocytes (control and +Astrocytes, respectively) and were assessed by western blotting (levels normalized to α-tubulin). Results are expressed and means of each group ± SD (*n* = 3 independent experiments); *p*-values were obtained by One-way ANOVA with Dunnett’s multiple comparison test vs. Ctrl P1D4 groups, NS = *p* > 0.05, * = *p* < 0.05, ** = *p* < 0.01, *** = *p* < 0.001.

**Figure 7:**
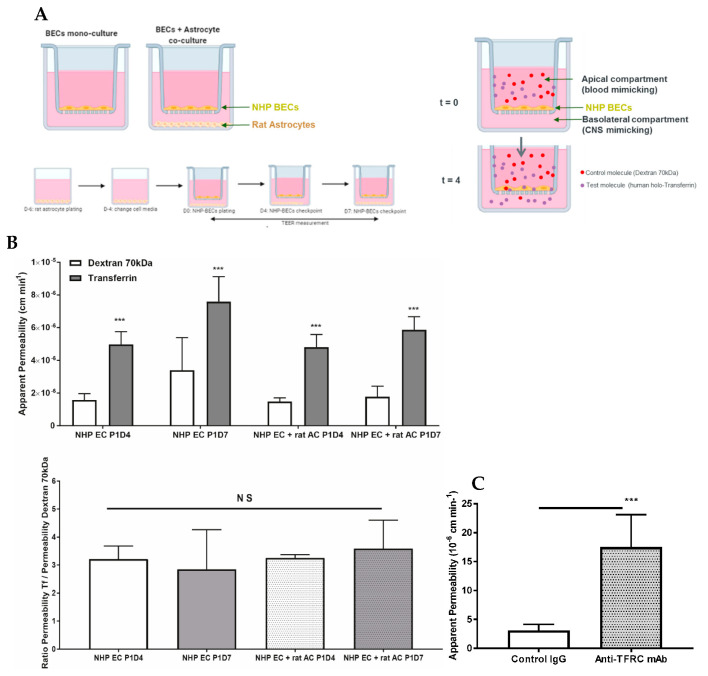
Proof of concept of the use of the present NHP-derived BBB model for evaluation of the apical to basolateral passage of selected molecules. (**A**) Schematic representation of the set-up of NHP-derived BBB model. NHP-derived BECs at P1 are plated on the insert, and rat astrocytes are optionally plated at the bottom of the well. Plating of rat primary astrocytes is performed 6 days before BECs are plated onto collagen and fibronectin-coated membrane filter inserts. This model is used at P1 following either 4 or 7 days in vitro. At the beginning of the assay, test molecules (human holo-transferrin and dextran 70 kDa) were incubated apically for 4 h at 37 °C. After 4 h, media from the apical and basolateral chambers were collected to assess paracellular flux (dextran 70 kDa) or transcytosis (transferrin). Such molecules present in the media from the apical and basolateral chambers were quantified by fluorescence or by IgG ELISA. (**B**) Permeability (10^−6^ cm min^−1^) from apical to basolateral passage of human holo-Transferrin (Tf) versus Dextran 70 kDa measured in the NHP BBB model. This model was used at P1 following either 4 or 7 days in vitro (P1D4 and P1D7, respectively) in the absence or presence of primary rat astrocytes (control and + rat AC, respectively). Results are expressed as mean ± S.D. (*n* = 3 independent experiments, performed in triplicate or quadruplicate). *p*-values were obtained by Two-way ANOVA with Sidak’s multiple comparisons test or One-way ANOVA with Dunnett’s multiple comparison test vs. NHP EC P1D4 group. NS: no statistical significance = *p* > 0.05, *** = *p* < 0.001. (**C**) Permeability (10^−6^ cm min^−1^) from apical to basolateral passage of an anti-TFRC antibody versus a control mouse IgG measured in the NHP BBB model. This model was used at P1 following either 4 days in vitro in the presence of primary rat astrocytes. Results are expressed as mean ± S.D. (*n* = 3 independent experiments, performed in quadruplicate). *p*-values were obtained by Student *t*-test NS: no statistical significance = *p* > 0.05, *** = *p* < 0.001.

Following puromycin purification in vitro during the P0 selection, NHP-derived BECs at P1 evidenced the formation of a tight monolayer, continuous and with growth inhibition at confluence. NHP-derived BECs at P1 in monoculture or co-cultured with rat astrocytes were set in culture for four (P1D4) or seven (P1D7) days for further molecular and functional characterization. Paracellular permeability of the endothelial layer was also monitored through TEER measurement, where monolayers in monoculture presented a maximal 140 Ω·cm^2^, while brain endothelial monolayers in co-culture with astrocytes evidenced a maximal TEER of 245 Ω·cm^2^ (Figure 6A). The expression of claudin5, ZO-1 and occludin proteins were found at the cell-cell junctions (Figure 6B). Time in culture or co-culture with astrocytes represented no changes in the protein expression in P1D7 vs. P1D4 regarding claudin5, ZO-1, and occludin (Figure 6C), except for VE-cadherin (1.4-fold decrease found by WB, *p* < 0.001, Figure 6C). TFRC-mediated transcytosis activity was Investig.igated by measuring the transport of human holo-transferrin from the apical to the basolateral compartment (Figure 7A), which showed to be significantly higher vs. Dextran 70 kDa, used as marker of paracellular passage (3.2-fold, *p* < 0.001, Figure 7B). TFRC-mediated transcytosis activity was statistically no different when NHP-BECs were co-cultured with or without astrocytes for seven days (P1D7). Similarly, the RMT transport of an internally developed antibody against the human and cynomolgus TFRC, was evaluated in the present model (co-culture with rat astrocytes, P1D4) vs. a control mouse IgG. A 5.7-fold higher apparent permeability was found for the anti-TFRC antibody vs. control mouse IgG (Figure 7C, *p* < 0.001). Taken together, these results indicate TFRC actively participates in RMT in the BEC-derived model.

## 4. Discussion

Decades Investig.ed in the search of CNS-active drugs have proven the formidable challenge of the BBB in the fight against diverse neurologic disorders. Primate studies may bring insights in the human BBB-focused research, as rhesus monkeys share greater transcriptomic similarities with human brains than mouse [83,84]. The NHP brain endothelium transcriptome is currently underrepresented, hampering a clearer elucidation of the primate BBB [10,85]. The establishment of the NHP-derived BBB model hereby presented constitutes a serious asset in comparison to cell line-based (hCMEC/D3, bEnd.3) and primary BBB models: (1) the starting material obtained from one animal is considerably greater and access to fresh post mortem tissue is much easier than for human, (2) BECs are obtained from freshly isolated post-mortem material, and therefore the BBB molecular features are well-preserved, (3) there is an evidently low variability between cultures from the same starting material, and (4) considerable retention of endothelial characteristics after several days in culture and cellular passaging. Furthermore, previous studies comparing isolated brain microvessels from rodents, primates and humans have reported consistent similarities between NHPs and humans from a molecular and pharmacokinetic standpoint, but significant differences have been retrieved between rodents and humans [10,66,86], favorizing the use of NHP or human-derived over rodent-derived BBB models as a tool in the prediction of CNS exposure of drugs and biologics in NHP and humans, as well as for target validation of novel drug targets at the primate level. On the other hand, commercially available human brain endothelial cells, such as hCMEC/D3 or human primary BECs, even though expressing some typical endothelial cell markers, they are reported to (1) have very low levels of expression of tight junctions, which are translated into low TEER values and relative high permeability towards small tracer molecules indicating paracellular leakiness, and (2) present major differences in the SLC and ABC transporter families and in diverse cell surface receptors, in comparison to freshly isolated brain microvessels, which have direct consequences when assessing transport mechanisms in these models [87]. As an alternatrive to the present model, PharmaCo-Cell Company has commercialized a BBB model prepared from primary cultures of monkey (*Macaca irus*) brain capillary endothelial cells, co-cultured with rat brain pericytes and astrocytes. However, its full characterization has not yet been published, with little information on how cells are obtained, and the model established. This newly generated dataset may therefore constitute a powerful tool for better understanding the BBB biology.

BECs constituting the BBB are in close contact with pericytes, encapsulated by the basement membrane, and surrounded by astrocytic endfeet processes and other cell types like microglia and neurons [88]. Consequently, the obtention of pure BECs, and thus of an accurate gene expression data on the BBB constitutes a real challenge. In the present study, brain microvessel isolation from a cynomolgus monkey brain was obtained through enzymatic digestion and gradient centrifugation, which yielded a highly enriched fraction of microvascular endothelial cells, but yet contaminated with pericyte, glial and circulating blood cell material. To eliminate any source of contamination, the isolated fraction was set in vitro and BECs selected through further exposure to puromycin [89].

Previously published comparisons of BBB gene expression profiles from freshly isolated rodent BECs versus primary cultures revealed evidence of dedifferentiation and/or down-regulation of BEC qualities (i.e., reduced levels of tight junction proteins, transporters and receptors) [75,90,91]. This is not surprising as cells cultivated in vitro are often at different stages of proliferation and differentiation. Nevertheless, we were willing to accept such limitations, as using an in vitro BBB model may yield some important insights into BEC response to external stimuli or stress, and for the prediction of passage of biotherapeutics. Nonetheless, the presented model was not thoroughly characterized at a molecular and functional level over P2, and therefore remains to be proven as an alternative model for high-throughput testing. Some authors have claimed the maintenance of barrier function properties, along with other phenotypic and cell purity properties in primary BECs following multiple passages [92,93], as inferred from the analysis of junction proteins (ZO-1 and β-catenin), endothelial cell markers (GLUT-1, vWF), and TEER measurement. However, it is very likely that more than one passage will be detrimental to the quality of the model, as documented in primary mouse models [94].

A key BBB feature is the presence of tight junctions between adjacent endothelial cells [95], where claudins (CLDN family), occludin (OCLN), and junctional adhesion molecules (JAMs) interact with cytoplasmic adaptors, including zona occludens and cingulin (CGNL1), establishing a linkage to the cytoskeleton and adherens junctions. In BECs, CLDN5 has shown to be the dominant CLDN family member and critical for the barrier formation and function [96,97]. The transcriptomic analysis hereby presented evidences CLDN5 as the highest expressed tight junction element within the cynomolgus brain vasculature, and likewise maintaining considerable high levels in vitro. A substantial expression following sub-culturing in vitro is also seen for other tight junctions, such as TJP1, OCLN, JAMs, and cadherins. The confirmation of protein expression of major tight junctions in our NHP-derived BECs, and notably their morphological distribution at the cell-to-cell interaction further hallmarks the tightness of the cell monolayer formed. Such results support that NHP-derived BECs maintain a relevant level of differentiation, representative of the brain microvasculature in its physiological state. The co-culture of BECs with astrocytes resulted in an increase on the average TEER measurements, but not in the global expression of tight junction and transporter proteins, findings corroborated by previous studies [98].

One of the most defining features of the BBB is the polarized expression of different transporters at the brain microvascular endothelium. The two major ABC transporters known at the BBB, ABCG2 and ABCB1, confirmed the highest expression levels among the ABC transporter superfamily in the NHP brain endothelium, a feature maintained in vitro, regardless of the downregulation observed for both transporters. These findings are consistent with reported transcriptomic and proteomic analyses [10,57,78], and evidence that ABCG2 greater expression over ABCB1 prevails among the primate BBB. Our analysis also confirmed ABCC4 as the third major efflux transporter present at the NHP BBB, corroborating previous studies [10,66,78]. The expression of several ABC transporters has been demonstrated to be species-dependent, where similar protein levels are found among rodents’ species, and among primates, but not from rodents to primates. The human [66], cynomolgus monkey [10], and marmoset [86] BBB’s are estimated to present 3–4-fold lower levels of ABCB1 than the rodent BBB. Likewise, consistent results regarding the expression levels of ABCC4 at the human and cynomolgus monkey BBB were also found [10,66], while the rodent BBB presents 5–8-fold higher abcc4 levels than those at the human and NHP BBB. Consequently, extrapolation of the BBB permeability obtained from rodent studies tends to underestimate the brain distribution of ABCB1 and ABCC4 substrates in human. In the present genomic analysis, we also identified the most abundant SLC transporters present at the primate BBB–SLC2A1, SLC7A5, SLC7A1, and SLC3A2, SLCO2B1, and SLC38A5, confirming previous findings [75,76,77,78]. These transporters remain the most expressed SLCs in vitro, except for SLC7A5 and SLC38A5 whose expressions considerably dropped in vitro. Therefore, SLC7A5 modest expression might compromise LAT-1 heterodimer activity in this model.

Our present study was also dedicated to exploring eventual differences of the vascular tree across regions of the brain, which is yet poorly addressed. In fact, regional specificities of the BBB have been reported in disease, injury [99,100,101,102], and aging [103], but to the best of our knowledge no report explores regional specificities of the BBB in the healthy brain. Given the circumventricular organs of the brain (e.g., posterior pituitary gland, pineal gland, median eminence of the hypothalamus) display a fenestrated endothelium with intercellular gaps [104], these were not included in the analyses. The present data suggest a considerable overlap of the molecular signatures of the brain endothelium obtained from the brainstem, cerebellum, cortex, hippocampus, and striatum, suggesting a rather homogeneous structure of the BBB, and therefore of its role across these brain regions. Nonetheless, these are important in the consolidation of our understanding regarding the heterogeneity of the BBB and how it may regulate brain function.

We also evaluated this NHP-derived BBB model in terms of RMT. Up to this date, TFRC remains the best validated RMT-mechanism allowing the enhancement brain exposure of biotherapeutics, widely reported by independent research groups [31,32,33,34,35]. Our study identified a TFRC-mediated transcytosis activity, as both transferrin and the anti-human/anti-cynomolgus TFRC were successfully transported from the apical to the basolateral compartment. Despite the downregulation of TFRC mRNA expression levels from isolated brain capillaries to P1, substantial expression levels are kept, which was also confirmed at protein level by WB. The present model presents improved tightness of the cell monolayer in comparison to cell line models such as hCMEC/D3, which displays low TEER values (~40 Ω·cm^2^, [3]) and for which an evaluation of the transcytosis of macromolecules across the model may only be carried using the so-called pulse-chase assay [105]. On the other hand, iPSC-derived BBB models have reported high TEER values (up to 2500 Ω·cm^2^, [106]), close to the ones reported in a physiological human BBB (~5000 Ω·cm^2^, [107]), however their differentiation conditions need to be carefully controlled to avoid an epithelial rather than endothelial phenotype [108,109]. In addition, no report can be found demonstrating transcytosis of anti-TfR antibodies in iPSC-derived BBB models, and comparable permeabilities for transferrin vs. human IgG have been shown [110].

The expression of other receptors participating in RMT was also confirmed by RNAseq in the present NHP-derived BBB model. Nonetheless, most RMT mechanisms reported to-date are ubiquitously expressed raising potential specificity and safety concerns. The identification of a novel RMT mechanism that is specifically and highly expressed at the BBB would represent a considerable asset in such competitive field, and the present transcriptomic analysis constitutes an important asset in the pursuit of such achievement. In fact, this NHP model probably represents one of the most adequate tools for the prediction of the human blood-to-brain permeability of macromolecules utilizing RMT mechanisms.

## Supplementary Materials

The following are available online at https://www.mdpi.com/1999-4923/12/10/967/s1, Figure S1: Transcriptional changes from P0D0 to P1D7 fractions, Figure S2: Transcriptional differences found across NHP brainstem, cerebellum, cortex, hippocampus and striatum, Figure S3: Schematic representations of the clathrin-mediated endocytosis and the caveolae-mediated endocytosis signaling pathways, and downregulation/upregulation prediction profiles, Figure S4: Expression of ABC and SLC transporters across P0D0, P0D7 and P1D7 fractions, Figure S5: ABC and SLC transporters positively correlated with the expression of PECAM1 across P0D0, P0D7 and P1D7 NHP cortical samples.
