# Supplementary material for: Non-Human Primate Blood–Brain Barrier and In Vitro Brain Endothelium: From Transcriptome to the Establishment of a New Model
Source: Pharmaceutics. 2020 Oct 14;12(10):967. doi: 10.3390/pharmaceutics12100967 (PMC7602447; doi:10.3390/pharmaceutics12100967)
Supplement: Supplementary file 1 [file pharmaceutics-12-00967-s001.pdf]

Supplementary Materials: Non-Human Primate Blood–Brain Barrier and In Vitro Brain Endothelium: From Transcriptome to the Establishment of a New Model

Catarina Chaves, Tuan-Minh Do, Céline Cegarra, Valérie Roudières, Sandrine Tolou, Gilbert Thill, Corinne Rocher, Michel Didier and Dominique Lesuisse

A

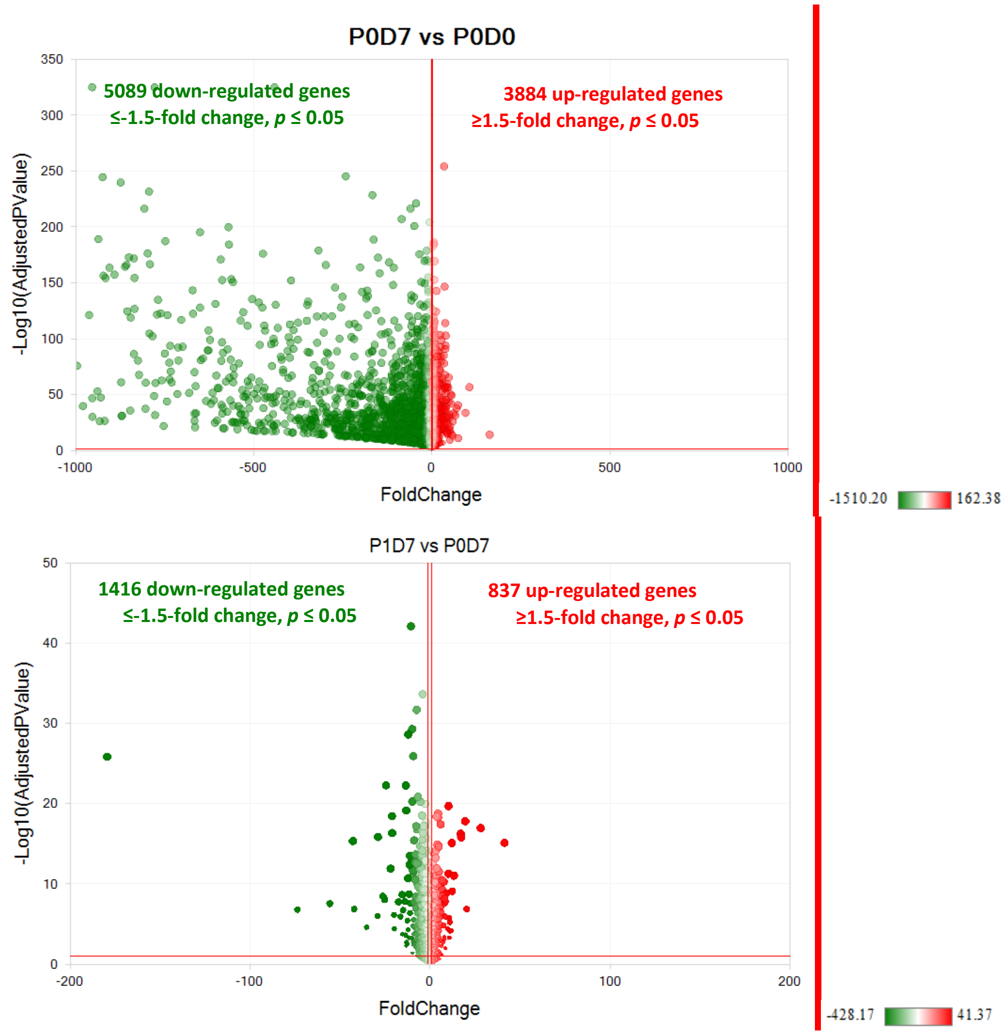

B

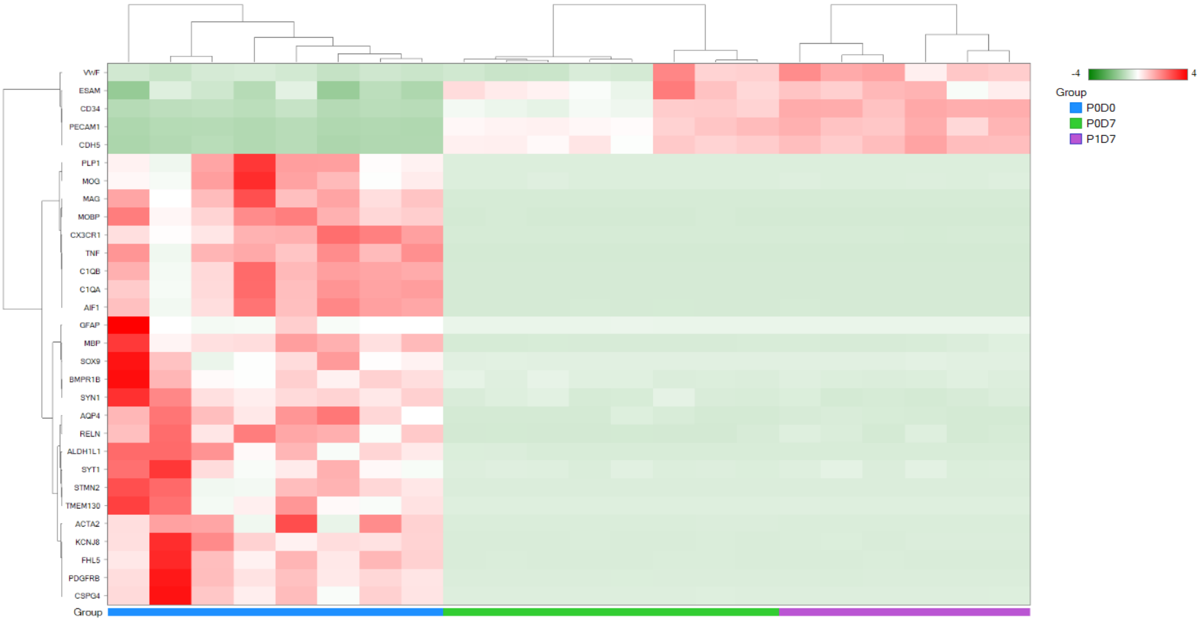

Figure S1. Transcriptional changes from P0D0 to P1D7 fractions. (**a**) Volcano plot as a representation of genes that are down-regulated and up-regulated in P0D7 in comparison to P0D0 EC population, and of P1D7 in comparison to P0D7 ECs (DESeq General Linear Model analysis, p value cutoff ≤ 0.05). (**b**) Heatmap plot representation of a hierarchical clustering of the FPKM variability of 5 cell-type specific genes across the P0D0 (n = 8), P0D7 (n = 8) and P1D7 (n = 6) groups. Data plotted using ArrayStudio, where variables were computed by applying a ward link and a correlation distance. Values subject to row centering and unit variance scaling.

A

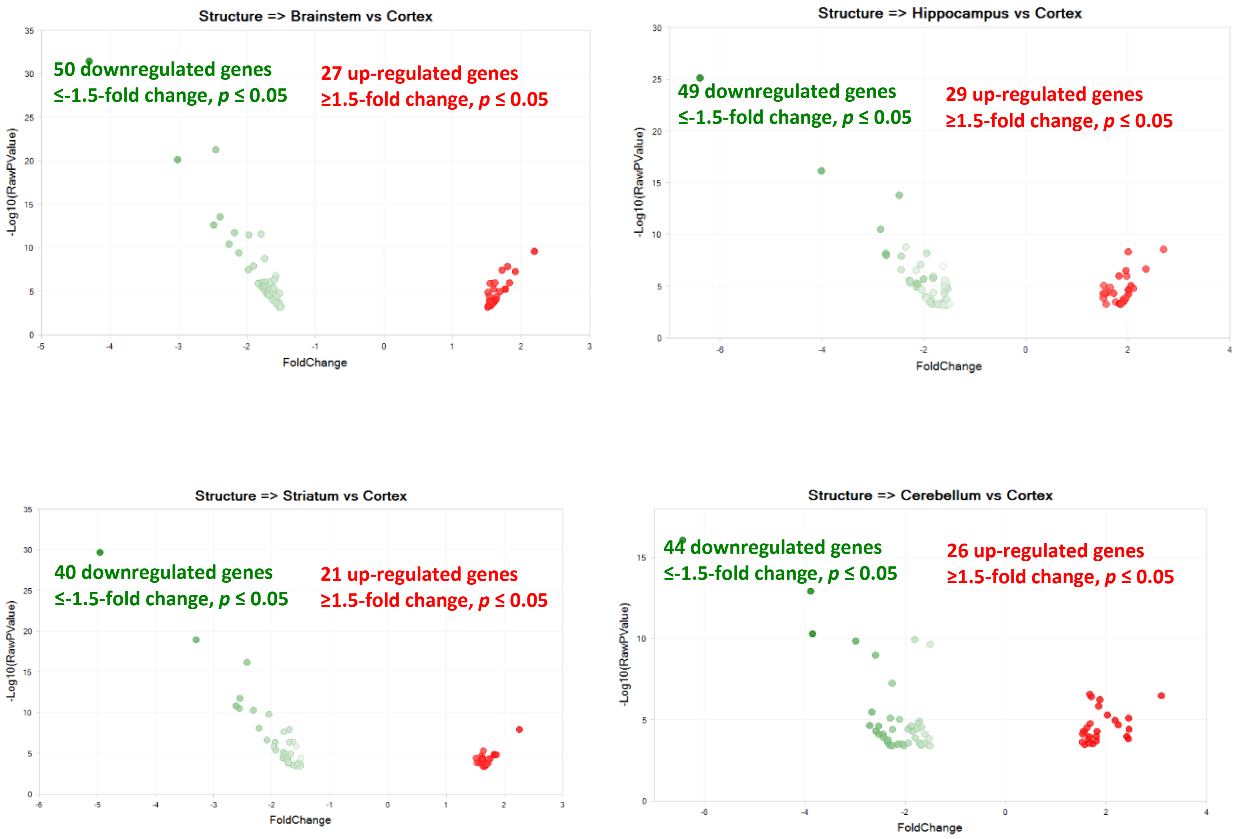

B

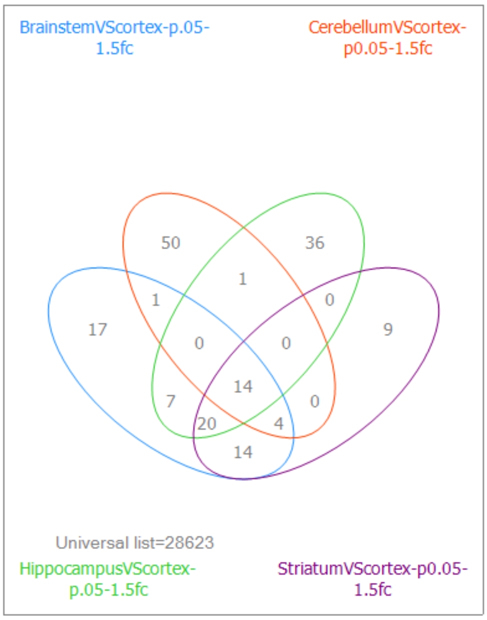

Figure S2. Transcriptional differences found across NHP brainstem, cerebellum, cortex, hippocampus and striatum. (**a**) Volcano plot as a representation of genes that are down-regulated and up-regulated in BECs from given brain structure *vs*. brain cortex (DESeq General Linear Model analysis, fold-change ± 1.5, p value cutoff ≤ 0.05). (**b**) Venn diagram representing the 173 genes found to be differentially expressed in BECs from four brain regions.

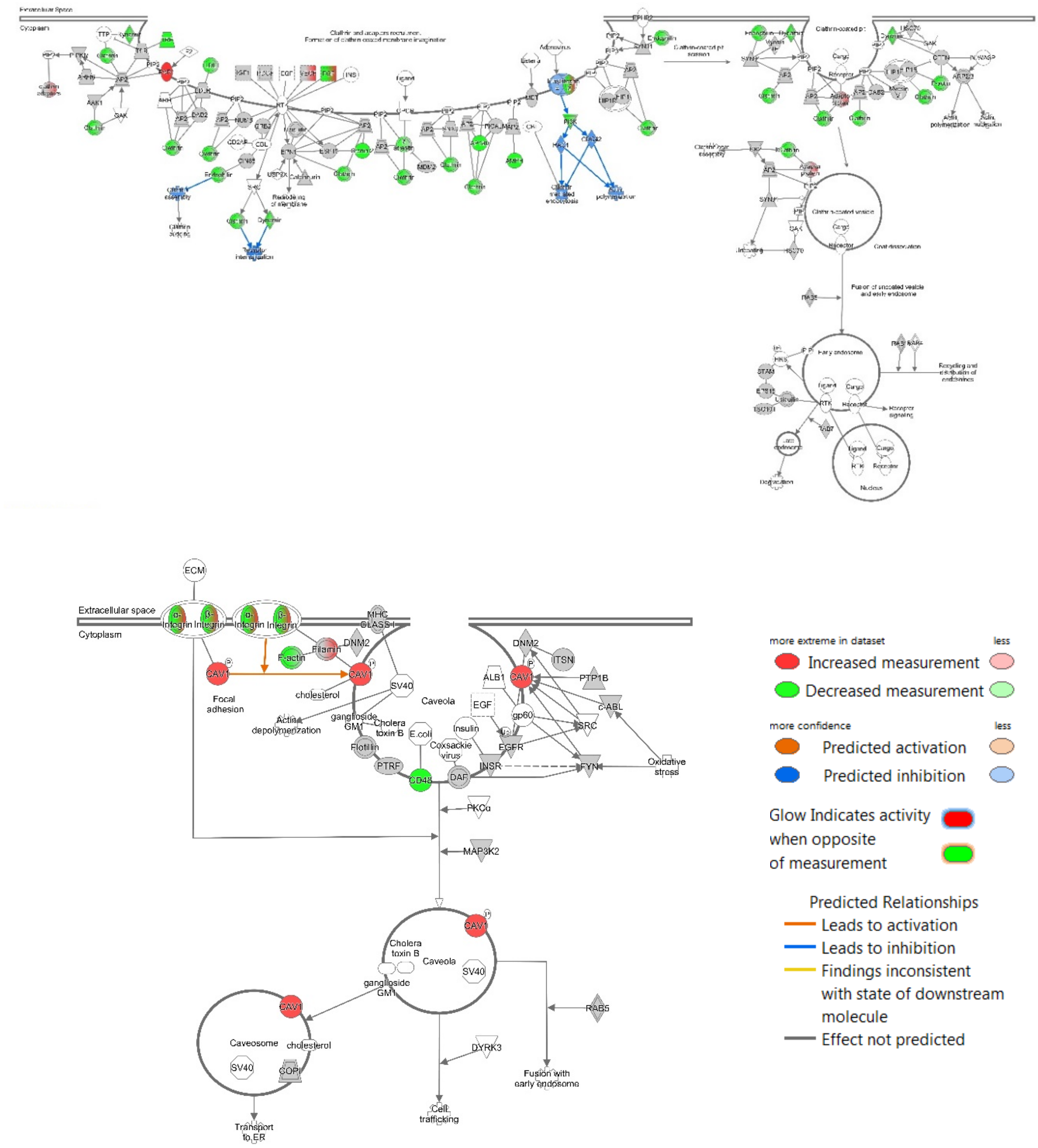

Figure S3. Schematic representations of the clathrin-mediated endocytosis and the caveolae-mediated endocytosis signaling pathways, and downregulation/upregulation prediction profiles. Expression patterns were compared from P1D7 to P0D0 samples. Upregulated elements are identified in red while downregulated elements are identified in green (see figure legend, color score from −10–10).

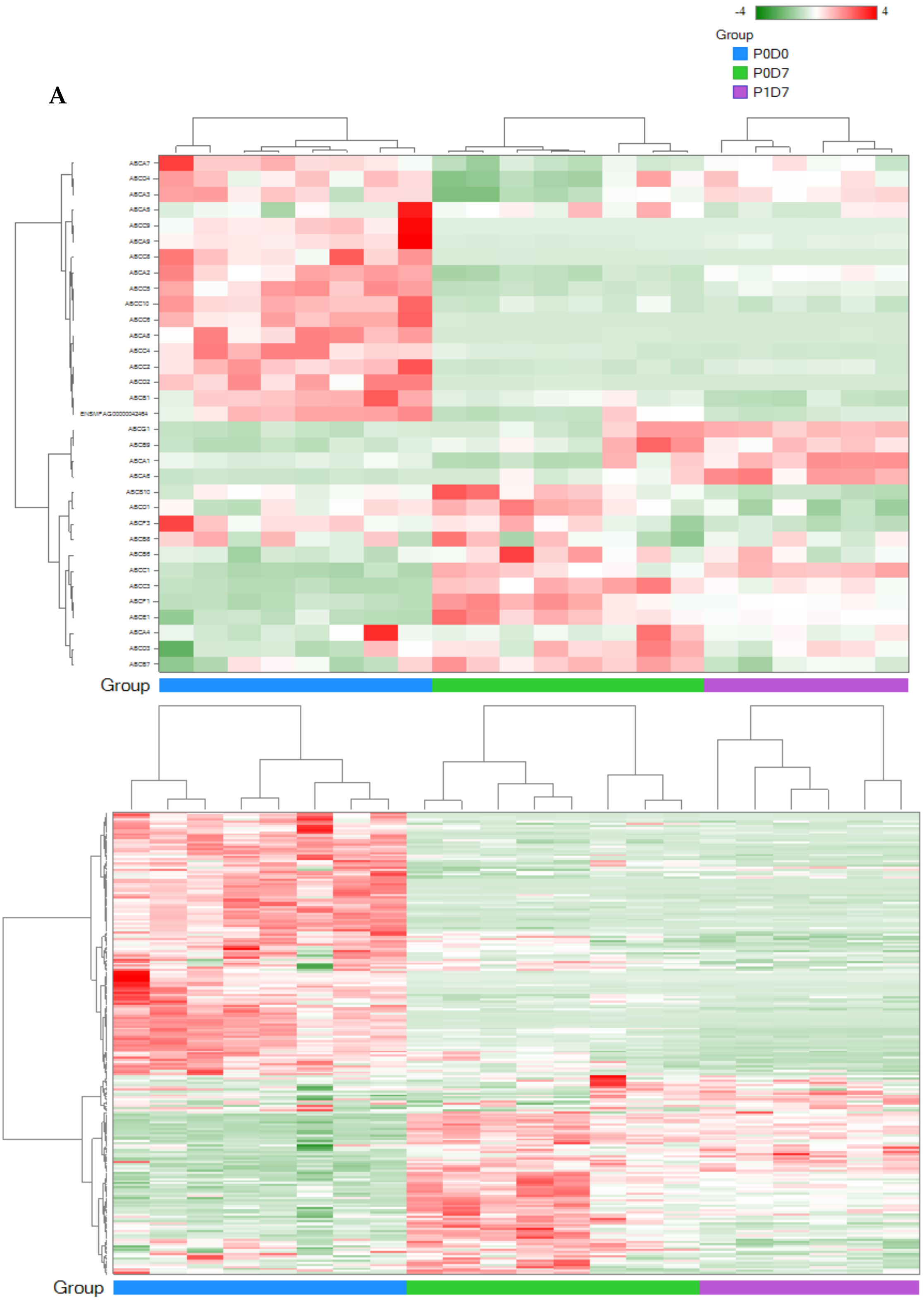

Figure S4. Expression of ABC and SLC transporters across P0D0, P0D7 and P1D7 fractions. (**a**) Heatmap plot representation of a hierarchical clustering of the FPKM variability of the 33 ABC transporters expressed genes across the P0D0 (n = 8), P0D7 (n = 8) and P1D7 (n = 6) groups. ABC transporter transcriptome changes are divided in two visible clusters: one cluster of genes is down-regulated in P0D7 and P1D7, while the second cluster shows a tendency for up-regulation in vitro in P0D7 and P1D7. Data plotted using ArrayStudio, where selected variables (33) were computed by applying a ward link and a correlation distance. Values subject to row centering and unit variance scaling. (**b**) Heatmap plot representation of a hierarchical clustering of the FPKM variability of the 216

SLC transporters expressed genes across the P0D0 (n = 8), P0D7 (n = 8) and P1D7 (n = 6) groups. SLC transporter transcriptome changes can be divided in two visible clusters: one cluster of genes is downregulated in P0D7 and P1D7, while the second cluster shows a tendency for up-regulation in vitro in P0D7 and P1D7. Data plotted using ArrayStudio, where selected variables (216) were computed by applying a ward link and a correlation distance. Values subject to row centering and unit variance scaling.

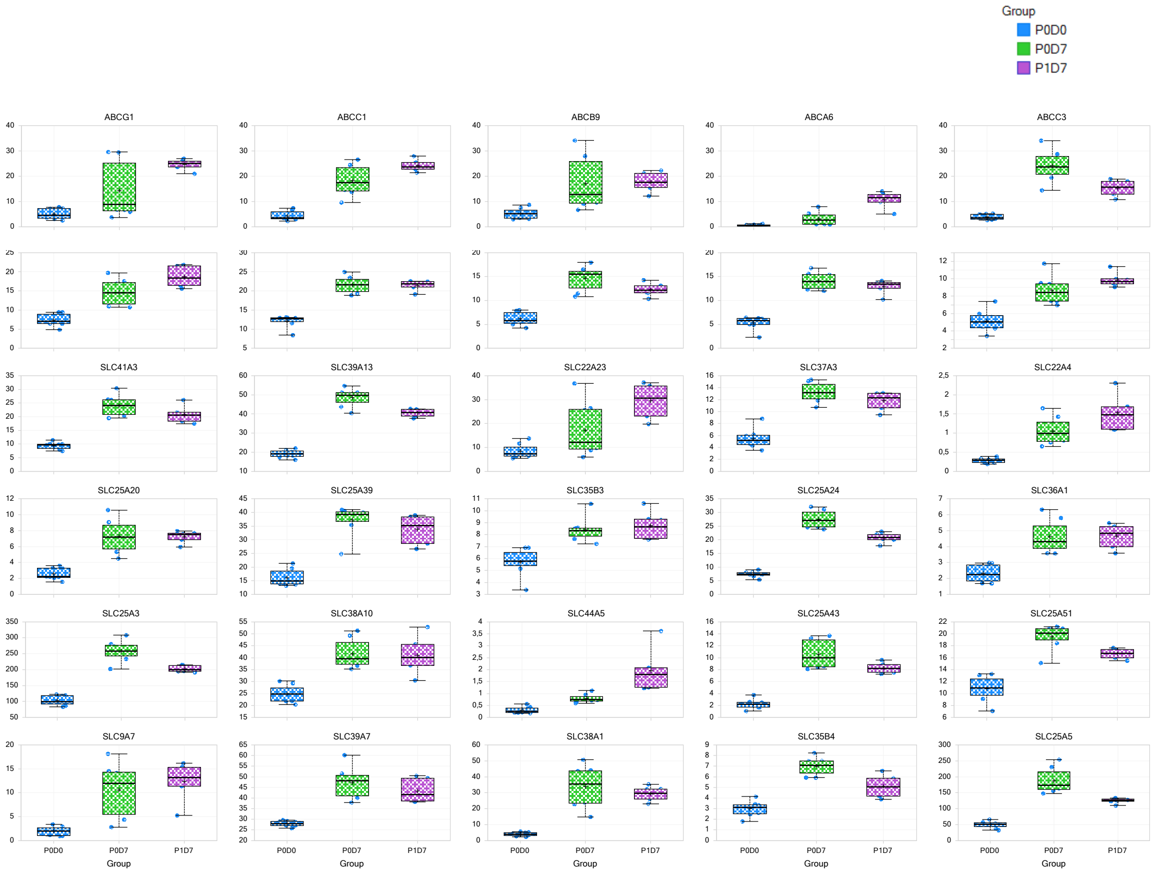

Supplementary Figure 5. ABC (5) and SLC (25) transporters positively correlated with the expression of PECAM1 across P0D0, P0D7 and P1D7 NHP cortical samples. Individual variable representation of PECAM1-correlated expression of ABC and SLC transporters in P0D0, P0D7 and P1D7 NHP-derived samples. Correlated genes were selected through a Pearson positive correlation with a p value < 0.001.
